# Promoting social capital, self-management and health literacy in older adults through a group-based intervention delivered in low-income urban areas: results of the randomized trial AEQUALIS

**DOI:** 10.1186/s12889-020-10094-9

**Published:** 2021-01-07

**Authors:** Sergi Blancafort Alias, Rosa Monteserín Nadal, Irene Moral, Marta Roqué Fígols, Xavier Rojano i Luque, Laura Coll-Planas

**Affiliations:** 1 Fundació Salut i Envelliment UAB. Casa Convalescència, Sant Antoni Maria Claret, 17, 4a planta, 08041 Barcelona, Spain; 2 Institute of Biomedical Research (IIB Sant Pau), Sant Quintí, 75-77, 08041 Barcelona, Spain; 3 Equip d’Atenció Sardenya, EAP Sardenya, Sardenya, 466, 08025 Barcelona, Spain

**Keywords:** Social medicine, Public health, Primary health care, Healthcare disparities, Aging, Self-management, Health literacy, Social capital

## Abstract

**Background:**

Evidence is scarce on how to promote health and decrease cumulative inequalities for disadvantaged older people. Downstream complex interventions focusing on intermediate factors (self-management, health literacy and social capital) may have the potential to mitigate the inequitable impacts of social determinants in health. The aim of the AEQUALIS study was to assess the effectiveness of a group-based intervention to improve self-perceived health as indicator of health inequality.

**Methods:**

Pragmatic randomised clinical trial addressed to older adults (≥ 60 years) living in urban disadvantaged areas with low self-perceived health. The intervention was delivered in primary care settings and community assets between 2015 and 2017 and consisted in 12 weekly sessions. The primary outcome was self-perceived health assessed in two ways: with the first item of the SF-12 questionnaire, and with the EQ-5D visual analog scale. Secondary outcomes were health-related quality of life, social capital, self-management, mental health and use of health services. Outcomes were assessed at baseline, post intervention and follow-up at 9 months after the end of the intervention.

**Results:**

390 people were allocated to the intervention group (IG) or the control group (CG) and 194 participants and 164 were included in the data analysis, respectively. Self perceived health as primary outcome assessed with SF-12-1 was not specifically affected by the intervention, but with the EQ-5D visual analog scale showed a significant increase at one-year follow-up only in the IG (MD=4.80, 95%CI [1.09, 8.52]). IG group improved health literacy in terms of a better understanding of medical information (− 0.62 [− 1.10, − 0.13]). The mental component of SF-12 improved (3.77 [1.82, 5.73]), and depressive symptoms decreased at post-intervention (− 1.26 [− 1.90, − 0.63]), and at follow-up (− 0.95 [− 1.62, − 0.27]). The use of antidepressants increased in CG at the follow-up (1.59 [0.33, 2.86]), while it remained stable in the IG.

**Conclusions:**

This study indicates that a group intervention with a strong social component, conducted in primary health care and community assets, shows promising effects on mental health and can be used as a strategy for health promotion among older adults in urban disadvantaged areas.

**Trial registration:**

ClinicalTrials.gov, NCT02733523. Registered 11 April 2016 - Retrospectively registered

**Supplementary Information:**

The online version contains supplementary material available at 10.1186/s12889-020-10094-9.

## Background

A number of factors are related to health inequities. It is well established that a low socioeconomic level is associated with poor health [1], with urban areas concentrating higher health inequalities, and ageing being also an axe of inequality [2]. Consequently, older people living in disadvantaged urban areas should be a specific focus for health equality research and policies, since they especially suffer its accumulative effects [3]. However, there is still a need for better evidence on how to address those inequalities [4]. Ndumbe-Eyoh & Moffat [5] classify interventions addressing health inequalities as “upstream” (structural and system-level changes), “midstream” (community or organizational level) and “downstream”. This latter include behavioural or psychosocial factors that occur at individual level and have the potential to mitigate the inequitable impacts of social determinants.

Health-related behaviours have been pointed as a core element to consider in interventions promoted by local primary health care agents [6]. An extensive body of evidence exists on the association between unhealthy behaviours and the burden of morbidity and mortality [7], as well as low health-related quality of life and mental illness [8, 9]. From a salutogenic perspective, the concept of positive mental health or mental well-being, which implies “feeling good”, has emerged as a strong predictor of overall health [10, 11]. Likewise, promising evidence correlates health-related behaviours with better self-management and mental well-being [12, 13].

Health literacy, is also strongly associated with health inequalities, for instance linking low socio-economic level with health choices related with worse outcomes [14]. Social capital, an umbrella concept that includes social support and participation in the community [15], has been identified as having both a buffer and a dependency effect on socioeconomic inequalities in health [16]. These factors are interrelated, as it has been suggested that older adults with low health literacy would be less prone to maintain a social network or make use of social resources [17]. Likewise, some authors have suggested that associations between health literacy and health behaviours are mediated by social cognitive factors such as self-efficacy [18]. Accordingly, these three modifiable intermediate factors (i.e., self-management, health literacy and social capital) might establish synergies with each other increasing their capacity to reduce health inequalities. Moreover, their synergies might reinforce older people’s wellbeing and might also be appropriate to addressed mental health.

When designing and evaluating interventions involving several interacting components, emerging methodologies that address complex interventions are valuable [19]. These methodologies also take in account dimensions of complexity such as the number and difficulty of behaviours required by those delivering or receiving the intervention, the number of groups or organisational levels targeted, the number and variability of outcomes and the degree of flexibility or tailoring of the intervention permitted. Another key question in evaluating complex interventions is how an intervention works. Hence, which are the active components within the intervention and how do they reach their effect [20]. As current evidence is weak on the effectivemess of policies and interventions to reduce health inequalities, more rigorous evidence-based approaches are needed to inform policymaking in this area [21]. Thus, the effectiveness of these complex interventions should be robustly assessed through randomised clinical trials [22].

To this end, we developed a complex intervention called “Sentir-nos Bé” (“Feeling well”) to promote self-management, health literacy and social capital in older adults living in disadvantaged urban areas in order to reduce health inequalities [23]. The main objective of the AEQUALIS study was to evaluate the effectiveness of the programme “Feeling well” to improve self-perceived health compared to usual care. Self-perceived health was chosen as a useful indicator since it correlates with general health status, mortality and morbidity, as well as health inequalities [24–26]. Secondarily, we assessed the effectiveness of the intervention in the improvement of health-related quality of life, social capital, self-management, health literacy, mental health and the use of health resources.

## Methods

The study protocol has been previously reported, and no relevant changes were made to the planned methods during the trial [23]. The trial is reported according to CONSORT 2010 statement [27].

### Study design, settings and patients

The trial had a pragmatic multicentric, parallel, individually-randomised controlled design with a 1:1 allocation ratio between intervention and control. Participants were recruited from people attending 16 primary care centers belonging to low-income neighborhoods. Selection of centers was based on convenience sampling. Individuals were considered eligible to participate in the study if: they were community-dwelling, aged 60 years or above, and perceived their health as fair or poor. Participants were excluded if: they needed help to go to the primary care center; had cognitive impairment or diagnosed dementia; had a medical condition that contraindicate physical activity; had any severe mental health problem that hinders participation in a group dynamic; or had an end of life situation.

### Randomisation and blinding

Once participants were included in the study, assigned an identification code, and completed the study baseline assessment, they were randomised to an intervention group (IG) or control group (CG). Concealed randomisation was conducted centrally at Fundació Salut i Envelliment UAB, using a computer-based random-block randomisation scheme, stratified by primary care centre. Participants and professionals conducting the group-based intervention remained unblinded. Blinding of outcome assessors was intended as assessments were conducted by professionals not involved with the intervention delivery or observation; however, this blinding was hard to sustain, as participants could easily reveal their group allocation.

### Interventions

The intervention of interest was a community program with multiple components, involving/targeting a broad range of outcomes, and new skills and behaviours to be acquired by those delivering and receiving the intervention. Thus, a complex intervention [19]. The intervention is described in accordance with the TIDieR guidelines [28]. It was aimed at promoting social support and participation (i.e., social capital), self-management and health literacy as intermediate factors between social determinants and health outcomes with potential to mitigate health inequalities. The intervention consisted in 12 sessions held weekly for 2 h and facilitated in groups up to 15 people. Nine of the 12 sessions were delivered in the primary care centre, and three consisted in local outings to public spaces to practice physical activity, a supermarket or a market, and a community equipment offering social activities that could be of interest to the participants. All group interventions were run between September 2015 and April 2017 and were facilitated by nine health and social care professionals (five nurses, two social workers and two general practitioners) previously trained as group facilitators by the research team on how to apply the intervention guide. At least one health or social care professional from each site participated as an observer of the intervention, completed an observation log including quantitative and qualitative measures of implementation such as fidelity and adherence. Participants in the control group were offered the intervention at the end of the study (delayed-intervention group).

### Outcomes

The primary outcome was self-perceived health, and it was assessed in two ways with the first item of the SF-12 questionnaire [29] (dichotomized into excellent-very good-good and fair-poor self-perceived health), and with the EQ-5D visual analog scale of the EuroQOL tool [30].

Secondary outcomes included health-related quality of life, self-management, health literacy, social capital (social support, social participation), mental health (emotional well-being, loneliness, depressive symptomatology, anxiolytics and antidepressants consumption), and use of health resources (visits to nurse, general practitioner, social worker, emergency units, and hospitalizations). The quality of life was measured by SF-12 questionnaire. Self-management was measured by the Appraisal of Self-Care Agency Scale (ASA-R) [31]. The Health Literacy Scale HLS-EU-16 [32] was used to measure some components of health literacy. Especifically, we estimated the difficulty in understanding medical information, finding out about activities that are good for mental well-being and assessing healthy lifestyles. Social support was measured by the Social Resources Inventory in Older Adults [33], and social participation was measured by Este II Subjective Social Participation Index [34]. Emotional well-being was measured by the Warwick-Edinburgh Mental Wellbeing Scale (WEMWBS) [35]. Loneliness was measured by the 11-item De Jong Gierveld Loneliness Scale [36], comprising subscales for emotional and social loneliness. Depressive symptomatology was measured by the Geriatric Depression Scale (GDS-5) [37]. Anxiolytics and antidepressants consumption, as well as use of health resources were obtained through electronic primary care records.

An English language version of the questionnaire used as part of the study design is included as a supplementary file. All outcomes were measured at the baseline (T0), after the intervention was completed (T1), and 9 months after the end of the intervention (T2). The use of health services (visits to nurse, general practitioner, social worker, emergency units, and hospitalizations) referred to the last 12 months, and were measured at T0 and T2. Assessments were conducted by health or social care professionals from the primary care centre not involved with the intervention delivery or observation. All outcome assessors received training in interview skills and outcome measure administration by the research group prior to the start.

### Sample size

A sample size of 390 participants was needed to detect a clinically relevant benefit in self-perceived health after 3-month intervention, defined as a 10% increase in the prevalence of participants who considered their health as good, according to the first item of SF-12. Details of computation have been previously published [23].

### Data analysis

First of all, a descriptive analysis was conducted to characterize the intervention and the control group regarding socio-demographic, health and psychosocial variables. Categorical variables were described as frequencies and percentages. Continuous variables were described as mean and standard deviation, or as medians and quartiles (Q1 and Q3) if they were clearly asymmetrical. Baseline comparability of the study arms was assessed by means of chi-square test for qualitative variables, and Student’s t-test and Wilcoxon tests for quantitative variables. In a second step, changes between baseline, post-intervention and 9 months after end intervention comparing study arms (experimental and control group) were carried out using multilevel mixed-effects models; linear, logistic or ordered logistics regressions were used when applicable. Repeated measures were considered nested at two levels, at center level and at individual level. It followed the intention-to-treat principle, thus including all randomized participants with a baseline assessment regardless of their permanence in the study or their loss to follow-up, withdrawal or drop-out. All statistical analyses used two-sided tests of statistical significance; estimates of the size of treatment effects were presented with confidence intervals and significance level. Significance levels were set to a 5% level. All analyses were performed with STATA/MP 14.0.

## Results

### Study participants and baseline characteristics

**Fig. 1:**
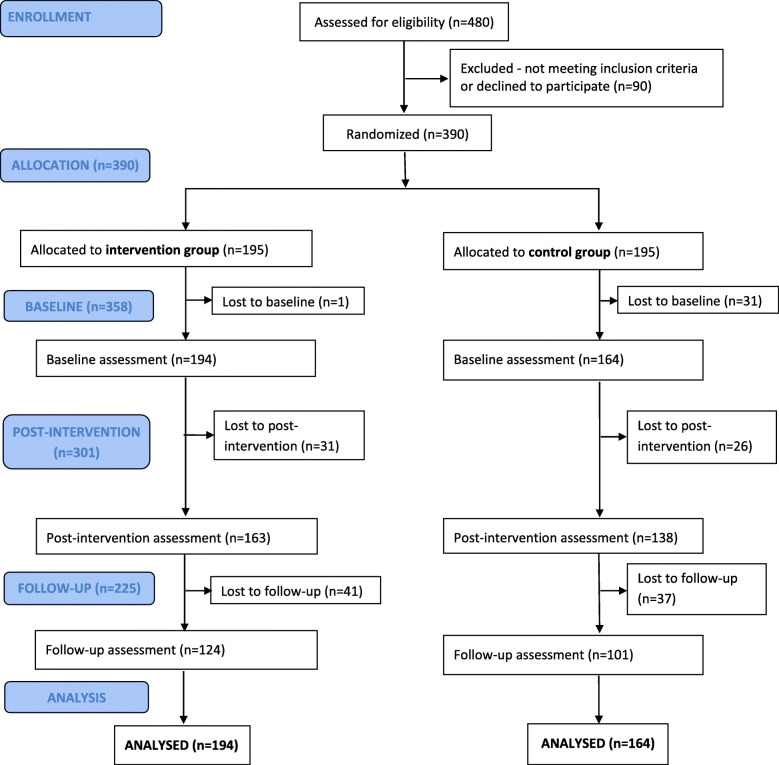
Flow chart of participants through each stage of randomized clinical trial

A total of 480 persons were screened for eligibility, of whom 90 were excluded because they did not meet inclusion criteria (*n*=52) or refused to participate (*n*=38). The 390 included participants were allocated to the IG (195 participants) or the CG (195 participants). 358 participants could be assessed at baseline (194 and 164), and included in the data analysis. Figure 1 illustrates the flow of participants through each stage of the study. Participants were recruited between 15/07/2015 and 31/01/2017. The last wave of 9 months follow-up assessments after the intervention ended on June 30th, 2018.

Descriptive values for each group of baseline socio-demographic, psycho-social and clinical characteristics of the study group participants are presented in Table 1. Participants were mostly women (81.4%) and their mean age was 73.6 ± 6.9 years. The majority of participants (82.2%) had received no formal education or completed only primary studies. Slightly more than a fifth (21.4%) were born in the same city where currently living. Four out of ten participants were widow (41.1%, mean of years of widowhood = 10.2) or lived alone (40.8%). The treatment and control groups were well balanced at baseline with regards to most socio-demographic, psycho-social and clinical characteristics. However in relation to CG, IG had on average 3.64 point less in mental health (SF-12 score on the mental component), a higher use of antidepressants (risk difference 15.54%) and 1.84 visits less to the nurse and more participants understanding medical information (risk difference 9.82%).

**Table 1:** Characteristics of participants at the baseline by treatment group

	Total (*n*=360)	Intervention group (*n*=194)	Control group (*n*=164)	*P* value
*Demographic characteristics*				
Women, *% (n)*	81.39 (293)	82.65 (162)	79.88 (131)	0.500
Age, *in years*, *mean (SD)*	73.63 (6.92)	73.41 (6.92)	73.88 (7.02)	0.531
Education level, *% (n) (without or only primary studies)*	82.23 (296)	84.18 (165)	79.88 (131)	0.548
Born in the same city where currently living, *% (n)*	21.39 (77)	23.47 (46)	18.90 (31)	0.301
Marital status				0.369
Widow, *% (n)*	41.11 (148)	44.39 (87)	37.20 (61)	
Years of widowhood, *median (Q1-Q3)*	7,8 (3–15)	6 (3–14)	10 (3–16.5)	
Same tabulation as "marital status" Living alone, *% (n)*	40.83 (147)	40.31 (79)	41.46 (68)	0.715
Same tabulation as "marital status" Caring for other people, *% (n)*	46.94 (169)	50.51 (99)	42.68 (70)	0.198
*Health status*				
Self-perceived health (SF-12-1)^a^, *% (n)*				0.291
Very good	0.28 (1)	0.51 (1)	0 (0)	
Good	2.22 (8)	1.02 (2)	3.66% (6)	
Fair	77.78 (280)	78.06 (153)	77.44% (127)	
Poor	19.72 (71)	20.41 (40)	18.90% (31)	
Self-perceived health (EQ-5D VAS)^b^, *mean (SD)*	51.31 (18.59)	49.97 (18.62)	52.89 (18.48)	0.140
SF-12^a^ Physical component, *mean (SD)*	36.49 (9.55)	36.59 (9.62)	36.36 (9.50)	0.829
SF-12^a^ Mental component, *mean (SD)*	40.04 (13.49)	38.38 (14.11)	42.02 (12.44)	0.013*
Self-management (ASA-R)^c^, *mean (SD)*	67.94 (12.06)	66.93 (12.07)	69.30 (11.97)	0.124
*Psychosocial characteristics*				
Social support				
Extension of social network^d^, *median (Q1-Q3)*	4 (3.5–5)	4 (3–5)	4 (4–5)	0.288
Emotional support^e^, *median (Q1-Q3)*	3 (2–4)	3 (2–4)	3 (2–4)	0.872
Instrumental support^e^, *median (Q1-Q3)*	1 (0–1)	1 (0–1)	1 (0–1)	0.421
Informational support^e^, *median (Q1-Q3)*	1 (0–2)	1 (0–2)	1 (0–2)	0.062
Social participation^f^, *% (n)*				0.950
Low	29.44 (106)	28.57 (56)	30.49 (50)	
Moderate	43.06 (155)	46.43 (91)	39.02 (64)	
High	21.94 (79)	19.39 (38)	25.00 (41)	
Same tabulation as "social participation" Emotional Wellbeing (WEMWBS)^g^, *mean (SD)*	53.78 (8.94)	52.93 (8.65)	54.91 (9.24)	0.135
Loneliness intensity^h^ (De Jong Gierveld Loneliness Scale), *% (n)*				0.508
Without loneliness	33.61 (121)	32.65 (64)	34.76 (57)	
Moderate	63.06 (227)	63.27 (124)	62.80 (103)	
Severe	2.50 (9)	3.57 (7)	1.22 (2)	
Very severe	0.56 (2)	0.51 (1)	0.61 (1)	
Same tabulation as "social participation" Depressive symptomatology (GDS-5)^i^, *% (n)*	57.22 (206)	59.69 (117)	54.27 (89)	0.505
Number of chronic diseases, *median (Q1-Q3)*	5 (3–8)	5 (3–8)	5 (3.75–8)	0.913
Number of chronic medications, *median (Q1-Q3)*	6 (4–8)	6 (4–8)	6 (4–8)	0.493
Anxiolytics consumption, *% (n)*	42.22 (152)	45.92 (90)	37.80 (62)	0.300
Antidepressants consumption, *% (n)*	30.28 (109)	37.24 (73)	21.95 (36)	0.006**
*Use of health services (last 12 months)*				
Visits to nurse, *median (Q1-Q3)*	5.5 (2–10)	5 (2–9)	6 (3–11)	0.088
Visits to general practitioner, *median (Q1-Q3)*	9 (5–13)	9 (5–13)	8 (5–13)	0.912
Visits to emergency units, *median (Q1-Q3)*	0 (0–1)	0.5 (0–2)	0 (0–1)	0.120

Boldface indicates between groups statistical significance at baseline

*P < 0.05

**P < 0.01

^a^SF-12: Short Form 12 Health Survey

^b^Visual Analog Scale of the EuroQOL: Range 1–100. Higher values indicate higher self-perceived health

^c^Appraisal of Self-Care Agency Scale (ASA-R): Range 24–120. Higher values indicate higher self-management

^d^Social Resources Inventory in Older Adults. Number of sources of relationship from 0 to 5 (partner, children, other relatives, neighbours and friends)

^e^Social Resources Inventory in Older Adults. Number of sources of relationship providing emotional/instrumental or informational support from 0 to 5 (partner, children, other relatives, neighbours and friends)

^f^Este II Subjective Social Participation Index

^g^Warwick-Edinburgh Mental Wellbeing Scale (WEMWBS). Higher scores indicate a higher level of mental well-being

^h^11-item De Jong Gierveld Loneliness Scale

^i^Geriatric Depression Scale (GDS-5). A score of 2 or more indicates depressive symptomatology

### Results of multilevel models on the effectiveness of the intervention over time

#### Primary outcome

**Fig. 2:**
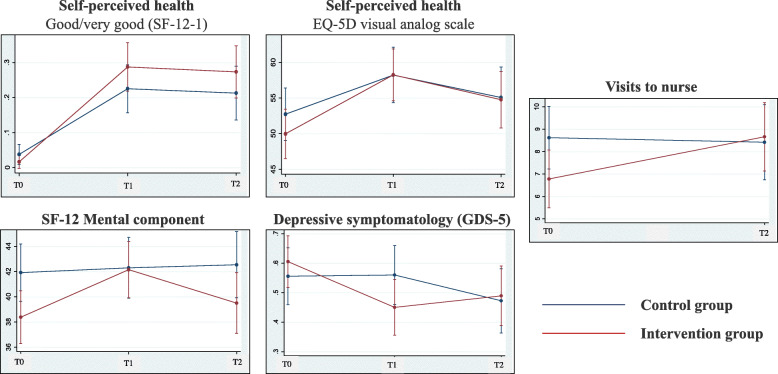
Average predicted values for outcomes by treatment and time of assessment

Between baseline and T1, good/very good self-perceived health assessed with SF-12 increased for participants of both groups: in the IG (mean change 5.15 [95%CI: 3.34, 6.97]) and the CG (3.27 [1.89, 4.66]). At follow-up, the improvement from baseline was maintained in both study arms, but the increase was higher for the IG with an absolute rise of 27.27% (5.01 [3.21, 6.82]), compared to a 16.14% (3.13 [1.71, 4.56]) in the CG (Table 2, Fig. 2). However, no differences between study groups were observed at any timepoint (Table 2).

**Table 2:** Adjusted predictions for outcomes by group allocation and time of assessment (*n* = 358)

	Baseline (T0)		Intra-group changes from T0 to T1		Intra-group changes from T0 to T2	
	Intervention	Control	Intervention	Control	Intervention	Control
**Primary outcome (Self-perceived health)**						
Excellent/Very Good/Good (SF-12-1)^a^, % (n)	1.53 (3)	3.66 (6)	5.15 (3.34, 6.97)**	3.27 (1.89, 4.66)**	5.01 (3.21, 6.82)**	3.13 (1.71, 4.56)**
EQ-5D visual analog scale^b^	49.97 (18.62)	52.89 (18.49)	8.29 (4.91, 11.67)**	5.49 (1.80, 9.18)**	4.80 (1.09, 8.52)*	2.34 (− 1.74, 6.43)
**Secondary outcomes**						
Health-related quality of life (SF-12)^a^						
Physical component, mean (SD)	36.59 (9.62)	36.3 (9.50)	1.57 (0.16, 2.97)*	2.95 (1.41, 4.50)**	1.22 (−0.34, 2.79)	1.36 (− 0.35, 3.08)
Mental component, mean (SD)	38.39 (14.11)*	42.03 (12.45)*	3.77 (1.82, 5.73)**	0.38 (−1.77, 2.53)	1.13 (− 1.05, 3.31)	0.62 (− 1.76, 3.01)
Self-management (ASA-R)^c^, mean (SD)	66.93 (12.07)	69.30 (11.97)	1.69 (−0.23, 3.62)	1.25 (−0.95, 3.44)	2.86 (0.77, 4.95)**	4.09 (1.72, 6.47)**
Health literacy (HLS-EU)						
Understand medical information, % (n)	25.51 (50)**	15.85 (26)**	−0.62 (−1.10, − 0.13)*	−0.45 (− 0.99, 0.08)	−0.25 (− 0.80, 0.29)	−0.32 (− 0.92, 0.29)
Find out about activities for mental well-being, % (n)	32.65 (64)	25.61 (42)	−0.45 (− 0.92, 0.02)	0.34 (− 1.17, 0.85)	−0.31 (− 0.82, 0.21)	0.18 (− 0.38, 0.74)
Assess healthy lifestyles, % (n)	17.35 (34)	14.03 (23)	−0.24 (− 0.72, 0.23)	−0.00 (− 0.51, 0.51)	−0.08 (− 0.60, 0.44)	0.09 (− 0.48, 0.66)
Social capital						
Extension of social network^d^, mean (SD)	3.95 (0.86)	4.04 (0.86)	−0.00 (− 0.09, 0.08)	−0.02 (− 0.12, 0.07)	−0.05 (− 0.15, 0.05)	0.00 (− 0.11, 0.11)
Low social participation^e^, % (n)	29.71 (52)	28.66 (45)	−0.23 (− 0.74, 0.28)	−0.08 (− 0.65, 0.49)	0.21 (− 0.35, 0.78)	−0.50 (− 1.14, 0.14)
Mental health						
Emotional well-being (WEMWBS)^f^, mean (SD)	52.93 (8.65)	54.91 (9.23)	1.49 (−0.01, 2.98)	0.11 (− 1.60, 1.82)	− 0.25 (− 1.86, 1.35)	0.08 (−1.84, 2.01)
Emotional loneliness subscale^g^, mean (SD)	2.63 (1.75)	2.53 (1.62)	−0.12 (− 0.35, 0.11)	−0.07 (− 0.32, 0.19)	−0.34 (− 0.60, − 0.08)*	−0.41 (− 0.70, − 0.13)**
Social loneliness subscale^g^, mean (SD)	1.22 (1.32)	1.15 (1.22)	0.12 (− 0.04, 0.29)	0.02 (− 0.16, 0.20)	−0.18 (− 0.36, 0.01)	−0.13 (− 0.34, 0.08)
Depressive symptomatology (GDS-5)^h^, % (n)	59.69 (117)	54.27 (89)	−1.26 (− 1.90, − 0.63)**	0.03 (− 0.64, 0.70)	−0.95 (− 1.62, − 0.27)**	−0.67 (− 1.42, 0.08)
Anxiolytics consumption, % (n)	45.92 (90)	37.80 (62)	−0.37 (− 1.16, 0.43)	0.27 (− 0.62, 1.16)	0.54 (− 0.34, 1.41)	−0.38 (− 1.40, 0.63)
Antidepressants consumption, % (n)	37.24 (73)**	21.95 (36)**	−0.20 (− 1.17, 0.77)	0.83 (− 0.31, 1.97)	1.05 (− 0.08, 2.17)	1.59 (0.33, 2.86)*
Use of health resources						
Visits to nurse, mean (SD)	6.88 (7.04)	8.72 (9.26)	N/A	N/A	1.72 (0.43, 3.02)**	−0.19 (− 1.64, 1.26)
Visits to general practitioner, mean (SD)	9.66 (5.55)	9.68 (6.03)	N/A	N/A	−0.36 (− 1.27, 0.54)	− 0.12 (− 1.13, 0.89)
Visits to emergency units, mean (SD)	1.24 (1.86)	0.91 (1.41)	N/A	N/A	−0.18 (− 0.55, 0.19)	0.31 (− 0.10, 0.72)

Boldface indicates between groups statistical significance at baseline

*P < 0.05

**P < 0.01

^a^SF-12: Short Form 12 Health Survey

^b^Visual Analog Scale of the EuroQOL: Range 1–100. Higher values indicate higher self-perceived health

^c^Appraisal of Self-Care Agency Scale (ASA-R): Range 24–120. Higher values indicate higher self-management

^d^Social Resources Inventory in Older Adults. Number of sources of relationship from 0 to 5 (partner, children, other relatives, neighbours and friends)

^e^Este II Subjective Social Participation Index

^f^Warwick-Edinburgh Mental Wellbeing Scale (WEMWBS). Higher scores indicate a higher level of mental well-being

^g^11-item De Jong Gierveld Loneliness Scale: subscales for emotional loneliness score 0–6 and social loneliness score 0–5. Higher scores indicate higher levels of loneliness

^h^Geriatric Depression Scale (GDS-5). A score of 2 or more indicates depressive symptomatology

Likewise, self-perceived health assessed with the visual analog scale also increased from T0 to T1 in both groups IG (8.29 [4.91, 11.67]) and CG (5.49 [1.80, 9.18]) (Table 2, Fig. 2) and no differences were found between study groups. Nevertheless, only the IG sustained the improvement until T2 (4.80 [1.09, 8.52]).

#### Secondary outcomes

When comparing the groups at T1, depressive symptomatology was higher in the CG compared to the IG (*P*=0.036). No further significant differences were found between the two study grups in the remaining secondary outcomes at this time point. When comparing changes between baseline assessments and T1, the physical component of the SF-12 improved in both groups: IG (1.57 [0.16, 2.97]) and CG (2.95 [1.41, 4.50]). However, only the IG improved the mental component (3.77 [1.82, 5.73]), showed higher levels of understanding medical information (− 0.62 [− 1.10, − 0.13]) and decreased depressive symptomatology (− 1.26 [− 1.90, − 0.63]). When comparing changes between baseline assessments and T2, higher levels of self-management were reported by both IG (2.86 [0.77, 4.95]) and CG (4.09 [1.72, 6.47]). Similarly, emotional loneliness decreased in both IG (− 0.34 [− 0.60, − 0.08]) and CG (− 0.41 [− 0.70, − 0.13]). The use of antidepressants increased significantly from 21.95 to 29.70% in the CG (1.59 [0.33, 2.86]), while it remained stable in the IG. Visits to nurse increased in the IG (1.72 [0.43, 3.02]) reaching a similar number of visits than the CG at baseline. Finally, when comparing changes between T1 and T2 assessments (results not shown in the table), social loneliness decreased in the IG (− 0.30 [− 0.49, − 0.11]). The decrease of depressive symptomatology observed at T1 was maintained in the IG until T2 (− 0.95 [− 1.62, − 0.27]).

## Discussion

This study assessed whether a complex intervention improved self-perceived health in older adults living in urban disadvantaged areas, and its impact on self-management, health literacy, social capital, mental health, health-related quality of life, and use of health resources. The primary outcome self perceived health assessed using SF-12-1 was not specifically affected by the intervention. Remarkably, the same outcome measured with the EuroQOL visual analog scale showed a significant increase at one-year follow-up only in the IG. Among secondary outcomes, the intervention seemed to improve health literacy in terms of understanding medical information and the mental component of quality of life, and to decrease social loneliness and depressive symptoms. It is also important to note that antidepressants consumption increased at the follow-up only in the CG, although we should be cautious in interpreting to what extent the programme prevents the prescription of antidepressants by addressing depressive symptoms with a non-medical intervention. Our research was unsuccessful in proving that the intervention had any effect on self-management, social capital, or visits to GP and emergency units. Unexpectadly, we found an increase in the visits to nurses in the IG, though it was also significantly lower at the baseline and reached the same levels as the CG.

The promising results reported by this study in mental health are supported by previous reseach which showed that the participation in a community-based intervention reduced depressive symptoms in older adults with comorbidity [38, 39]. Similarly, multi-domain interventions among community-living older persons and in primary care settings also reduced depressive symptomatology and demonstrated a significant improvement in mental health [40, 41]. The lack of results in self-management and social capital differ from previous studies [42] which shown that strengthening social support in group-based interventions might improve self-management behaviours among socioeconomically deprived patients. In contrast, other researchers have reported that financial constraints and low socioeconomic status are barriers to effective self-management [43, 44]. The use of existing social support groups has been also underlined as an acceptable and attractive method of delivering a self-management intervention to older people in socio-economically disadvantaged areas [45]. The results obtained in health literacy are in line with those reported from similar group-based interventions [14]. Finally, there are several possible explanations for the unexpected results in the use of health resources. Previous findings [46], showed a decrease in visits to the general practitioner in a group-based intervention targeting older people suffering from loneliness. Regarding the higher number of visits to the nurse, other studies in the same context [47] found the same result and suggested that it might be due to greater trust with these professionals as a consequence of their role as observers of the intervention, or due to a higher awareness of health problems by participants thus increasing indeed the number of appropriate visits.

### Strengths and limitations

To the best of our knowledge, this is the first randomised clinical trial addressing health inequalities by promoting self management, health literacy and social capital and showing promising effects on mental health. It is plausible that some limitations might have influenced the results obtained. Firstly, both grups differed at baseline in the mental component of SF-12, understanding medical information, antidepressants consumption and number of visits to the nurse. However, randomization is the most robust method of preventing the selection bias that occurs when the group which receive the intervention differ systematically from the control group, in ways likely to affect outcomes [48, 49]. Another possible source of bias is the high loss to follow-up specially in the control group. Researchers have shown that recruitment and retention of older adults in clinical trials is usually a challenging task due to their comorbidities as well as social and cultural barriers, especially in low-income contexts [50, 51]. Hence, further research should address challenges such as drop-out and lack of adherence as a common shared problem with other group interventions of health promotion in primary care settings, specially in deprived socio-economically contexts and older people. Thirdly, a major source of contamination comes from the fact that participants could easily revealed their group allocation and thus blinding outcome assessors were hard to sustain, and thus it might lead to biased estimates of treatment effect. Finally, as the main eligibility criteria of the study, self-perceived health could be misleading in terms of recruitment, given that health may have different meanings for older people and professionals. Our hypothesis is in line with previous results [52] that considers this indicator of great importance for the design of programmes aimed at improving older’s people health.

We feel strongly that the study also provides a novel approach in terms of the participating actors, the problems tackled and the strategy applied. This reinforces previous findings in the literature [53] and fairly supports the idea that a community-based collaboration with primary care providers can improve health strategies, and would appear to indicate that linking organized primary health care with community-based interventions may be a promising direction for research and practice. This strategy might be especially relevant considering the increasing workload of primary care professionals with the growing proportion of older people with chronic diseases [54]. In this sense, practical effectiveness has been appointed as a key issue when assessing a complex intervention [55]. Finally, this intervention can guide initiatives aimed to improve a perception of health which is based on their strengths and needs and goes beyond the chronic disease, and be transferred to other settings and become a useful resource to which health professionals may refer older people, specially those suffering psychosocial problems. To this aim, professionals from 16 primary care centers were trained as potential facilitators of the intervention, and the Institut Català de la Salut (Catalan Institute of Health), an organization dependent on the Health Department of the Catalan Government, commissioned the research team to train during 2019 and 2020 nearly a hundred professionals from Barcelona urban area.

## Conclusions

The results of this study provide evidence to inform policy makers how to promote health among older adults in urban disadvantaged areas while addressing inequity. It has shown how a group intervention with a strong social component, conducted in primary health care and community assets, can lead to promising effects on mental health and may be used as a model for its biopsychosocial approach on health, with an emphasis on salutogenesis by means of promoting self-management, health literacy and social capital.

## Supplementary Information

**Additional file 1.**

## Data Availability

The full trial protocol can be accessed in:https://bmcpublichealth.biomedcentral.com/articles/10.1186/s12889-018-5219-x#Bib1 The datasets and materials used and/or analysed during the current study are available from the corresponding author on reasonable request.

## Abbreviations

IG: Intervention group
CG: Control group
MD: Mean difference
CI: Confidence interval
